# Outcomes of Concomitant Atrial Fibrillation Ablation and Left Atrial Appendage Closure: A Retrospective Single-Center Experience

**DOI:** 10.1016/j.jacadv.2024.101377

**Published:** 2024-11-14

**Authors:** Ashraf Alzahrani, Oussama M. Wazni, Ayman Hussein, Chadi Tabaja, Rehan Karmali, Himanshu Sajja, Benjamin Klein, Saqer Alkharabsheh, Arwa Younis, Joseph Sipko, Astefanos Al Dalakta, Alveena Syed Batool, Eoin Donnelan, Shady Nakhla, Bryan Baranowski, Mandeep Bhargava, Thomas Callahan, Thomas Dresing, Pasquale Santangeli, David Martin, John Rickard, Koji Higuchi, Jakub Sroubek, Wael A. Jaber, Samir R. Kapadia, Tyler Taigen, Walid Saliba, Mohamed Kanj

**Affiliations:** a Division of Cardiovascular Medicine, Department of Internal Medicine, University of Iowa Hospitals and Clinics, Iowa City, Iowa, USA; b Section of Clinical Cardiac Electrophysiology, Department of Cardiovascular Medicine, Heart, Vascular and Thoracic Institute, Cleveland Clinic, Cleveland, Ohio, USA; c Section of Cardiology, Department of Internal Medicine, Tulane University School of Medicine, New Orleans, Louisiana, USA

**Keywords:** atrial fibrillation, catheter ablation, device occlusion, left atrial appendage closure, WATCHMAN

## Abstract

**Background:**

Catheter ablation is an effective therapy in the management of atrial fibrillation (AF). Left atrial appendage closure (LAAC) is an alternative to anticoagulation for stroke prevention in patients with bleeding risks.

**Objectives:**

The purpose of this study was to assess the safety and efficacy of combining AF ablation and LAAC in a single procedure.

**Methods:**

This retrospective observational study included consecutive patients who underwent concomitant AF ablation and LAAC between June 2015 and August 2021. We assessed the safety and efficacy of the combined procedure with respect to procedural success, periprocedural complications, thromboembolism, bleeding, and arrhythmia recurrence.

**Results:**

A total of 178 patients (mean age 72.1 ± 8.7 years, 60.7% male) were identified. The mean CHA_2_DS_2_VASc and HAS-BLED scores were 4.0 ± 1.4 and 2.8 ± 1.1, respectively. Pulmonary vein isolation was achieved in all patients. LAAC was aborted in 15 cases (success rate of 91.6%). The periprocedural complications rate was 6.2%. The median follow-up duration was 412 days IQR: 213 to 781 days. There were 2 strokes and 3 transient ischemic attacks, equating to an annual risk of 1.7% at 1 year and 4.6% at 2 years. Complete seal was achieved in 97.5% intraprocedurally and in 73.7% on initial follow-up, with no major leaks identified. There were 2 cases (1.3%) of device-related thrombus that resolved with anticoagulation. Thirty-four bleeding events occurred in 28 patients (17.4%). Anticoagulation was discontinued in 93.8% of patients.

**Conclusions:**

Concomitant AF ablation and LAAC could be considered in appropriate patients in centers of clinical expertise.

Although catheter ablation is an effective therapy in the management of atrial fibrillation (AF), its efficacy in stroke prevention remains controversial.^1^, ^2^, ^3^ Moreover, post-ablation anticoagulation discontinuation, either due to side effects or self-discontinuation, is not uncommon.^4^, ^5^, ^6^, ^7^ Current consensus recommendations address this issue and recommend using the CHA_2_DS_2_-VASC score as a basis for continuing or discontinuing oral anticoagulation (OAC) regardless of ablation outcome.^8^

Left atrial appendage closure (LAAC) has emerged as an alternative to anticoagulation for stroke prevention in patients at elevated bleeding risk.^9^^,^^10^ The Prospective randomized evaluation of the Watchman Left Atrial Appendage Closure device in patients with atrial fibrillation versus long-term warfarin therapy: the PREVAIL trial^9^ and Percutaneous Left Atrial Appendage Closure vs Warfarin for Atrial Fibrillation (PROTECT AF) trial^10^^,^^11^ evaluated the role of the WATCHMAN device (Boston Scientific) in LAAC. A meta-analysis^12^ of these two trials and continued access registry data have shown that WATCHMAN implantation led to a reduction in hemorrhagic stroke, cardiovascular death, and rates of bleeding. The PINNACLE FLX trial^13^ evaluated the safety and effectiveness of the WATCHMAN FLX device and demonstrated higher efficacy and a lower incidence of adverse events.

Given the procedural similarities between LAAC and AF ablation, including venous access, anesthesia, and transseptal puncture, interest has grown in recent years in combining both procedures. This concomitant procedure could result in less risk exposure for patients and reduced health care resource utilization. Although a few observational studies in Europe and China investigating the combined ablation approach have shown promising initial results, the limited sample sizes underscore the critical need to gather and report more data to better substantiate the evidence behind this procedure. To date, there are no United States-based reports in the literature. As such, we are reporting our experience with the concomitant procedure.

We aimed to assess the safety and efficacy of combining AF ablation and LAAC in a single procedure with respect to procedural success, periprocedural complications, thromboembolism, bleeding, and arrhythmia recurrence in a large patient series.

## Methods

### Study design

This was a retrospective, observational, and descriptive study conducted at a large quaternary medical center.

### Data collection

All patients undergoing AF ablation and WATCHMAN implant at our institution are enrolled in a prospectively maintained registry for procedural profiles, complications, and outcomes. Data were collected from this registry and supplemented by chart review of the electronic medical record. This included provider documentation from outpatient cardiology appointments, emergency department visits, admissions, documented telephone encounters, imaging reports, cardiovascular testing reports, and procedural notes. Variables were transferred to a standardized abstraction form on an encrypted Microsoft Excel file for analysis. Data collection was nonblinded and will be made available from the corresponding author upon reasonable request.

### Subjects

The patient sample was collected from our registry and included all patients who underwent the concomitant procedure between June 2015 and August 2021 at our institution based on appropriate use criteria (Supplemental Table 1).

### Procedural details

All patients were started on a direct oral anticoagulant (DOAC) within 3 to 4 weeks before the procedure. Under general anesthesia, transesophageal echocardiography (TEE) was performed to exclude left atrial appendageal (LAA) thrombus and obtain LAA measurements. The TEE was then pulled back into the proximal esophagus, and an esophageal temperature probe was inserted (CIRCA).

Femoral venous sheaths were placed using ultrasound guidance. Heparin was initiated before transseptal access and titrated to target ACT 300-400s. Using intracardiac echocardiography and fluoroscopic guidance, one or two transeptal punctures were performed at the performing physician’s discretion with at least one of the transseptal punctures suitable for optimizing LAA closure.

Eleven patients had cryoablation, and 167 patients had radiofrequency ablation.

Radiofrequency ablation was performed to isolate the pulmonary veins and posterior wall using a Thermocool Smarttouch catheter (Biosense Webster).

Cryoablation was performed using ArcticFront catheter (Medtronic Inc). Two 3-4-minute occlusive cryoballoon applications were administered to each pulmonary vein (PV). Additional applications were performed to achieve isolation. Phrenic nerve pacing was performed continuously for all right-sided PV cryoablations.

After confirming PV isolation (entrance and exit block), a sheath more suitable for LAA closure was exchanged with the WATCHMAN delivery sheath. TEE was performed to reobtain LAA measurements. A LAAC device was advanced and deployed under TEE guidance, and after confirming the position, anchor, size, seal criteria,^11^ the device was released.

Following LAA closure, intracardiac echocardiography and TEE were reviewed for any pericardial effusion. All catheters were removed, and long sheaths were withdrawn to the inferior vena cava. Heparin was discontinued and protamine was administered. Hemostasis was obtained using manual compression, collagen plugs, or figure-of-8 sutures.

### Postprocedural management

All patients were discharged with a trans-telephonic arrhythmia monitor unless they had an implantable cardiac monitor and were instructed to record a 30-second telemetry strip weekly or when symptomatic during the initial 3-month follow-up. For patients who had atrial tachyarrhythmias and those who had symptoms suggestive of arrhythmias within the initial 3 months, monitoring was extended beyond that period. Follow-up visits were scheduled at 3, 6, and 12 months after ablation and every 6 months thereafter.

Postprocedure, DOAC and low-dose (81 mg) aspirin were administered for the intended 60 to 90 days, with almost all patients remaining on this combination for this time frame (149 patients at 60 days; 113 patients at 90 days). Patients were scheduled for a follow-up appointment that included TEE at 60 days and/or cardiac computed tomography (CT) at 90 to 120 days post-implant. On evidence of adequate LAA seal by TEE or CT (and with no plans of further ablations), patients were directed to discontinue DOAC therapy at 60 to 90 days and begin a dual antiplatelet therapy regimen of clopidogrel (75 mg) plus low-dose aspirin until 6 months post-implant. Adequate LAA seal was defined as the absence of device-related thrombus (DRT), peri-device leak (PDL) >5 mm, or uncovered lobes. If the seal was inadequate, patients continued DOAC plus aspirin and were re-evaluated at 6 months post-implant.

### Statistical analysis

Descriptive statistics were used to summarize baseline data, including number (percentage) for categorical data and mean ± SD for numerical data. A *P* value <0.05 was considered statistically significant. Statistical analyses were performed using the R Studio software, version 1.1.463.

### Definitions

PDL was defined as the presence of flow past or around the edge of an implanted device into the fundus of the LAA, as determined by TEE or CT. A major PDL was defined as >5 mm, and a minor PDL as ≤5 mm.

Stroke was defined as an infarction of central nervous system tissue (brain, spinal cord, or retinal cells) attributable to ischemia, based on neuropathologic, neuroimaging, and/or clinical evidence (*ie*, persistence of symptoms or findings) of permanent tissue injury as assessed by a neurologist at the time of presentation.

Transient ischemic attack (TIA) was defined as a transient episode of neurologic dysfunction caused by focal brain, spinal cord, or retinal ischemia, without acute infarction. This was assessed by a neurologist at the time of presentation and corroborated with neuroimaging.

Arrhythmia recurrence was defined as electrocardiographic documentation of AF, atrial flutter, or sustained atrial tachycardia lasting >30s that was either self-reported by the patient, assessed by a medical provider, or captured on electrocardiogram or telemetry strips. If the exact date of recurrence was unknown, then the date of the first mention of an arrhythmia recurrence in the medical record was used.

Major bleeding events were defined per the Bleeding Academic Research Consortium (BARC)-3 or BARC-5 guidelines (a summary can be found in the Supplemental Appendix).

### Ethics

The study was approved by the Cleveland Clinic Foundation Institutional Review Board.

## Results

### Demographics

A total of 178 patients were scheduled to undergo concomitant AF ablation and LAAC procedures between June 2015 and August 2021. The mean age at the time of the procedure was 72.1 ± 8.7 years, and 60.7% were male. The mean left ventricular ejection fraction was 52.9% ± 10.4%. The mean CHADS_2_ and CHA_2_DS_2_VASc scores were 2.2 ± 1.1 (median 2; IQR: 1-3) and 4.0 ± 1.4 (median 4; IQR: 3-5), respectively. The mean HAS-BLED score was 2.8 ± 1.1 (median 3; IQR: 2-4). Baseline characteristics of the study population are listed in Table 1.

**Table 1:** Baseline Characteristics

Age (y)	
Mean ± SD	72.1 ± 8.7
Range	24-93
Sex	
Male	108 (60.7%)
Female	70 (39.3%)
CHADS_2_ score	
Mean ± SD	2.2 ± 1.1
Range	1-5
Median (IQR)	2 (1-3)
CHA_2_DS_2_-VASc score	
Mean ± SD	4 ± 1.4
Range	1-8
Median (IQR)	4 (3-5)
HAS-BLED score	
Mean ± SD	2.8 ± 1.1
Range	0-6
Median (IQR)	3 (2-4)
Components of CHADS_2_ and CHA_2_DS_2_-VASc scores	
Congestive heart failure	57 (32%)
Hypertension	144 (80.9%)
Age ≥75 y	66 (37.1%)
Diabetes	47 (26.4%)
History of TIA/stroke	40 (22.5%)
Vascular disease	82 (46.1%)
Age 65–74 y	92 (51.7%)
Female	72 (40.4%)
Components of HAS-BLED scores	
Uncontrolled hypertension	82 (46.1%)
Abnormal renal function	31 (17.4%)
Abnormal liver function	5 (2.8%)
History of ischemic or hemorrhagic stroke	36 (20.2%)
Prior major bleeding or predisposition to bleeding	107 (60.1%)
Labile international normalized ratio	7 (3.9%)
Concomitant use of drugs	63 (35.4%)
Alcohol abuse	9 (5.1%)
Age >65 y	156 (87.6%)
Known duration of atrial fibrillation (months)	
Mean ± SD	74 ± 71.2
Median (IQR)	54 (20-113)
Range	0-404
Atrial fibrillation pattern	
Paroxysmal	84 (47.2%)
Persistent	70 (39.3%)
Long-standing persistent	24 (13.5%)
Left ventricular ejection fraction (%)	
Mean ± SD	52.9 ± 10.4
Range	15-72
Prior cardioversion	121 (68%)
Prior ablation	66 (37.1%)
Type of oral anticoagulant	
Direct oral anticoagulant (DOAC)	142 (79.8%)
Vitamin K antagonist (VKI)	29 (16.3%)
None	6 (3.4%)
Enoxaparin	1 (0.6%)
On class 1 or 3 anti-arrhythmic drug (AAD)	70 (39.3%)
AAD type	
Class 1	12 (6.7%)
Class 3	58 (32.6%)

Values are mean ± SD, range, n (%), or median (IQR).

AAD = anti-arrhythmic drug; TIA = transient ischemic attack.

AF was paroxysmal in 84 patients (47.2%), persistent in 70 patients (39.3%), and long-standing persistent in 24 patients (13.5%). The mean duration of known AF was 74.0 ± 71.2 months (range: 0-404 months, median 54 months, [IQR]: 20 to 113 months). Most patients had at least one prior direct current cardioversion (n = 121, 68.0%), and 66 patients (37.1%) had a prior ablation. Most patients (96.6%) were on OAC before the procedure, with 142 (79.8%) on a DOAC, 29 (16.3%) on warfarin, and one on enoxaparin (0.6%). There were 70 patients (39.3%) on a class 1 or class 3 anti-arrhythmic drug before the procedure, with 12 (6.7%) on a class 1 agent and 58 (32.6%) on a class 3 agent.

LAAC indications were gastrointestinal bleeding (n = 51, 28.7%), recurrent falls risk (n = 33, 18.5%), intracranial or subarachnoid hemorrhage (n = 17, 9.6%), anemia (n = 11, 6.2%), and epistaxis (n = 11, 6.2%). The remaining indications can be found in the Supplemental Table 1.

### Procedural data

A total of 102 patients (57.3%) underwent LAAC with the legacy WATCHMAN device, and 76 patients (42.7%) with the WATCHMAN FLX device. Of the total 178 patients, 163 (91.6%) successfully underwent concomitant AF ablation and LAAC. All unsuccessful cases (n = 15, 8.4%) were due to aborting the LAAC portion of the procedure. Reasons for aborting LAAC included unsuitable anatomy in 12 patients, LAA sludge or thrombus that appeared after the ablation procedure in 2 patients, and significant edema at the Coumadin ridge in 1 patient.

**Figure 1:**
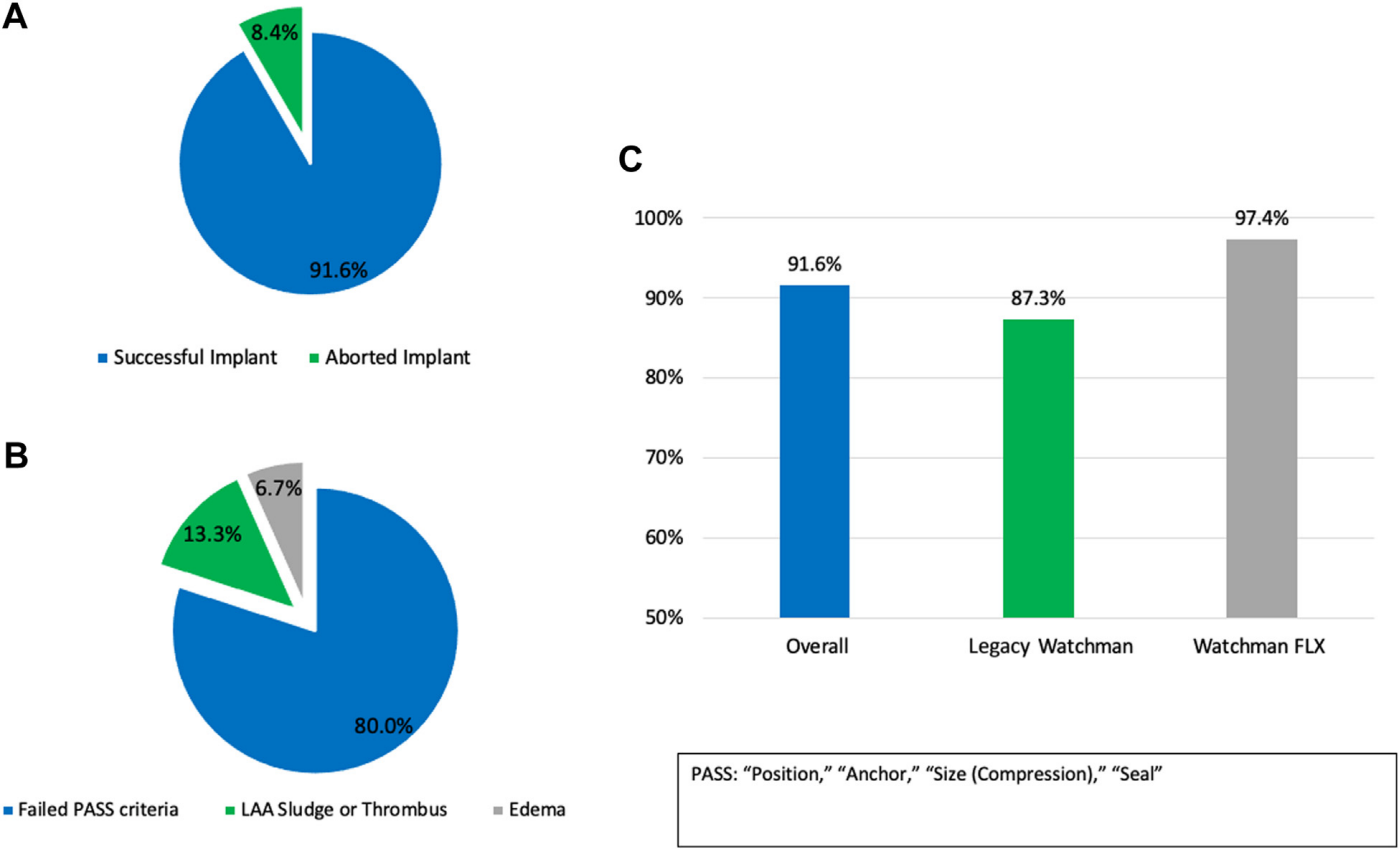
Procedural Success (A) Overall procedural success rate. (B) Reasons for aborting the LAAC portion. (C) Procedural success rate stratified by device type. LAA = left atrial appendage.

When evaluating the success rate according to device type, 89 of 102 patients (87.3%) in the legacy WATCHMAN group underwent successful placement, and 74 of 76 (97.4%) patients in the WATCHMAN FLX group underwent successful placement. The procedural success rate stratified by device type and reasons for aborting the LAAC portion are in Figure 1.

The mean LAA maximum diameter was 22.2 ± 3.9 mm. There was no significant change in the maximum diameter before or after ablation. The most common device size used was the 27 mm device. The mean minimum and maximum compression rates were 19.0% ± 5.4% and 24.6% ± 6%, respectively. There were 44 cases (27.0%) requiring at least one recapture and 19 cases (11.7%) requiring at least one device size change. After the procedure, 159 patients (97.5%) achieved complete seal, and four patients (2.5%) had a PDL ≤5 mm (Table 2). The mean procedural time was 177.2 ± 64 minutes, and the mean fluoroscopic time was 21 ± 14 minutes (Central Illustration).

**Table 2:** Procedural Data

Outcome	Summary Statistic
Ablation energy type	
Radiofrequency	168 (94%)
Cryoablation	11 (6%)
Device implant success rate	
Successful implant^a^	163 (91.6%)
Aborted implant	15 (8.4%)
Device type	
Watchman	102 (57.3%)
Watchman FLX	76 (42.7%)
Success rate according to device type	
Successful Watchman	89/102 (87.3%)
Successful Watchman FLX	74/76 (97.4%)
Reason for aborting	
Anatomic difficulty	12/178 (6.7%)
LAA sludge or thrombus	2/178 (1.1%)
Edema	1/178 (0.6%)
Procedural time in minutes	177.2 ± 64
Fluoroscopic time in minutes	21 ± 14
Device size (mm)	
Mean ± SD	27.3 ± 3.7
Median	27
Mode	27
Left atrial appendage diameter (mm)	
Mean ± SD	22.2 ± 3.9
Compression	
Compression not documented	14 (8.6%)
Minimum compression	19 ± 5.4
Maximum compression	24.6 ± 6
Maximum compression 10%-20%	46 (28.2%)
Maximum compression 21%-30%	82 (50.3%)
Maximum compression >30%	21 (12.9%)
Seal	
Complete	159/163 (97.5%)
≤5 mm leak	4/163 (2.5%)
>5 mm leak	0/163 (0%)
Recaptures	
Cases requiring at least 1 recapture	44 (27%)
# of partial recaptures	27
# of complete recaptures	37
Device size change	
Cases requiring at least 1 device size change	19 (11.7%)
# of device size changes	22
Periprocedural complications	11 (6.2%)
Pericarditis	5/178 (2.8%)
Vascular access site injury	3/178 (1.7%)
Perforation/tamponade	2/178 (1.1%)
Periprocedural stroke	1/178 (0.6%)

Values are n (%), n/N (%), or mean ± SD.

LAA = left atrial appendage.

a Out of the 163 successfully implanted cases, 2 cases had subsequent left atrial appendage perforation in the postprocedural period requiring surgical intervention and removal of the LAAC device.

**Central Illustration:**
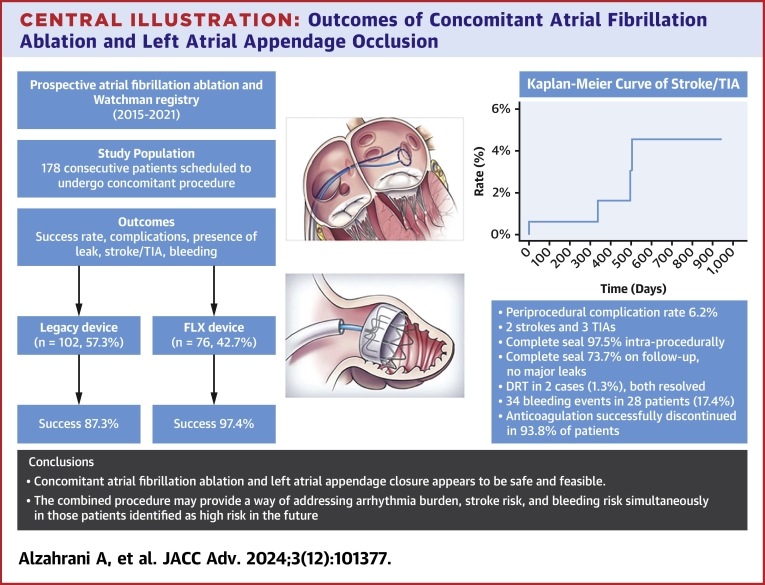
Outcomes of Concomitant Atrial Fibrillation Ablation and Left Atrial Appendage Occlusion DRT = device-related thrombus; TIA = transient ischemic attack.

### Periprocedural complications

The overall periprocedural complication rate was 6.2%. Two patients (1.1%) suffered an LAA perforation and tamponade in the immediate postprocedural period requiring surgical intervention. The first case was a legacy WATCHMAN device where part of the device metal pierced the LAA. The device was retrieved and the LAA was sutured. The second device was a WATCHMAN FLX. There was a laceration of the LAA tip, which was sutured, and the device was kept in place. There were 3 (1.7%) vascular access site injuries, with 1 (0.6%) groin hematoma, 1 (0.6%) retroperitoneal hematoma, and 1 (0.6%) arteriovenous (AV) fistula. One patient (0.6%) experienced a periprocedural stroke. This patient, who had symptomatic persistent AF and a history of stroke and LAA thrombus, had previously failed treatment with warfarin (INR 3-5) and multiple DOACs. At the time of the procedure, the patient was on subcutaneous low-molecular-weight heparin. Notably, the patient was noted to have LAA sludge and low flow during LAAC that cleared following the administration of isoproterenol. The stroke was recognized 140 minutes after the end of the procedure. A summary figure is in the Supplemental Figure 1.

### Initial imaging

Of the remaining 161 successfully completed concomitant AF ablation and LAAC procedures and with the LAAC device in place (excluding the two patients with immediate perforation described above), 152 patients (94.4%; n = 83 legacy WATCHMAN, n = 69 WATCHMAN FLX) had follow-up imaging, with 144 (89.4%) undergoing TEE, and 8 (5.0%) undergoing CT scan. The mean time to follow-up imaging was 85.4 ± 70.3 days, median 63.5 days ([IQR]:50-101.3 days). There were 112 patients (73.7%) with a complete seal, 40 patients (26.3%) with a PDL ≤ 5 mm, and no patient showed a PDL >5 mm. Of these, 25 minor PDLs were with the legacy device, and 15 PDLs were with the FLX device. When the ≤5 mm leaks were further categorized by size, 25/40 were <3 mm and 10/40 were 3-5 mm. The most common PDL location was anterior (32.5%). Findings are summarized in Table 3.

**Table 3:** Follow-Up Imaging Data

Outcome	Summary Statistic
Imaging completion	
Completed any form of imaging	152/161 (93.3%)
Completed TEE after procedure	144/161 (88.3%)
Completed CT scan instead of TEE	8/161 (4.9%)
Time lapse between procedure and TEE	
Mean ± SD	85.4 ± 70.3
Median (IQR)	63.5 (50-101.3)
Mode	50
TEE within 90 d of procedure	91 (55.8%)
TEE within 120 d of procedure	123 (75.5%)
TEE after 120 d	53 (32.5%)
Seal	
Complete seal	112/152 (73.7%)
≤5 mm leak	40/152 (26.3%)
<3 mm leak	25/152 (16.4%)
3-5 mm leak	10/152 (6.6%)
Unspecified minor leak size	5/152 (3.3%)
>5 mm leak	0 (0%)
Seal stratified by device	
Legacy Watchman	83
Complete seal	58/83 (69.9%)
≤5 mm leak	25/83 (30.1%)
>5 mm leak	0 (0%)
Watchman FLX	69
Complete seal	54/69 (78.3%)
≤5 mm leak	15/69 (21.7%)
>5 mm leak	0 (0%)
Atrial septal defect present	38/152 (25%)
Device-related thrombus	2/152 (1.3%)

Values are n/N (%), mean ± SD, or median (IQR).

CT = computed tomography; TEE = transesophageal echocardiogram.

**Figure 2:**
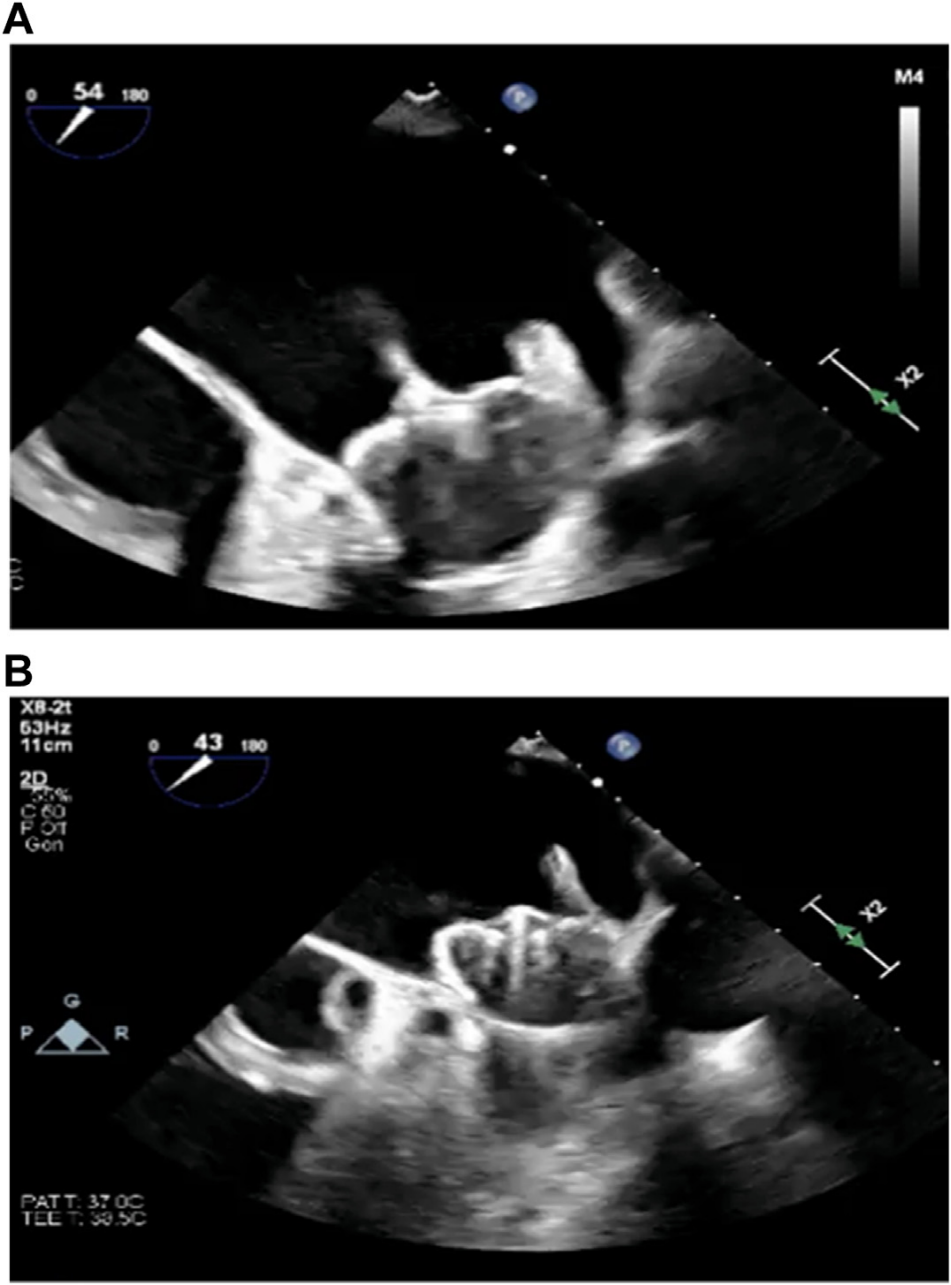
Device Tilting Seen With Significant Coumadin Ridge Edema at the Landing Zone (A) TEE at the time of device implant. (B) TEE at 95 days post-implant. TEE = transesophageal echocardiogram.

There were no device dislodgments. However, there were 2 cases of device tilting, one resulting in a 2 mm leak (Figure 2). One had cryoablation, and the other had radiofrequency ablation. Significant edema on the Coumadin ridge was a common feature in both cases.

There was persistent left-to-right flow across the atrial septum in 38 patients (25.0%). DRT was reported in 2 cases (1.3%) (Supplemental Figure 2).

### Follow-up

The mean total follow-up time was 553.3 ± 448.0 days with a median of 412 days (IQR: 213-781 days). A summary of the follow-up data can be found in Table 4.

**Table 4:** Patient Follow-Up

Outcome	Summary Statistic
Last known status	
Alive	141/161 (87.6%)
Deceased	20/161 (12.4%)
Duration of follow-up (d)	
Mean ± SD	553.3 ± 448
Range	8-2,132
Median (IQR)	412 (213-781)
Cause of death	
Cancer	4/161 (2.5%)
Unknown	4/161 (2.5%)
Cardiac arrest	3/161 (1.9%)
Hemorrhage	2/161 (1.2%)
ADHF	2/161 (1.2%)
ICH	1/161 (0.6%)
Complication of cardiac surgery	1/161 (0.6%)
Acute limb ischemia	1/161 (0.6%)
IPF	1/161 (0.6%)
Sepsis	1/161 (0.6%)
MVA	1/161 (0.6%)
Pulmonary embolism	1/161 (0.6%)
Pneumonia	1/161 (0.6%)
COPD	1/161 (0.6%)
Thromboembolic	
Stroke	2/161 (1.2%)
TIA	3/161 (1.9%)
Pulmonary embolism	1/161 (0.6%)
Deep vein thrombosis	1/161 (0.6%)
Hemorrhagic	
Gastrointestinal bleeding	16/161 (9.9%)
Genitourinary bleeding	5/161 (3.1%)
Epistaxis	5/161 (3.1%)
Intracranial hemorrhage	2/161 (1.2%)
Cutaneous hematoma	2/161 (1.2%)
Hemarthrosis	1/161 (0.6%)
Arrhythmia recurrence	
Recurrence after blanking	63/157 (39.1%)
Cardioversion	
# of patients requiring DCCV within blanking	20/161 (12.4%)
# of patients requiring DCCV after blanking	25/157 (15.5%)
# of DCCVs	74
Ablation	
Repeat ablation	11/161 (6.8%)
Atrioventricular nodal ablation	2/161 (1.2%)
Medication	
OAC discontinued	151/161 (93.8%)
AAD discontinued^a^	38/62 (61%)
Repeat imaging	
Repeat TEE beyond initial postprocedure TEE	21/161 (13%)
Seal	
Complete	15/21 (71.4%)
<5 mm leak	6/21 (28.6%)
>5 mm leak	1/21 (4.8%)
Device thrombosis	0 (0%)

Values are n/N (%), mean ± SD, median (IQR), or n.

AAD = anti-arrhythmic drug; ADHF = acute decompensated heart failure; ICH = intracranial hemorrhage; IPF = idiopathic pulmonary fibrosis; COPD = chronic obstructive lung disease; OAC = oral anticoagulant; MVA = motor vehicle accident; other abbreviations as in Tables 1 and 3.

a At baseline, 70 patients were on AADs out of the whole pool of patients. Of the patients who completed the procedure successfully and were followed longitudinally, 62 were already on AADs and 9 were started on AADs during follow-up. Overall, 38 patients had their AAD discontinued.

### Thromboembolism

During the follow-up period, one additional stroke occurred, bringing the total number of strokes to 2 (1.2%). In addition, there were 3 TIAs (1.9%). The stroke occurred 505 days postprocedure, whereas the TIAs occurred 339, 498, and 951 days after the procedure, respectively. All these patients were not on OAC and were on aspirin monotherapy at the time of stroke. All patients had a complete seal at the time of the event. Two TIAs occurred with the FLX device, whereas two strokes and 1 TIA were with the legacy device. None of the patients had an arrhythmia recurrence at the time of stroke/TIA. Table 5 summarizes the characteristics of the strokes/TIAs.

**Table 5:** Summary Characteristics of Patients Who Developed Stroke or TIA

#	Event	Time to Event	Age	Sex	CHADS-VASC	HAS-BLED	Device Type	Seal	OAC	Antiplatelet	Presentation	Management	Other	Arrhythmia Recurrence
1	Stroke	505 d	71	Male	5	3	Legacy 33 mm	Complete	None	Aspirin 81 mg	R MCA stroke	Started DOAC + aspirin	Residual ASD present	No
2	TIA	951 d	73	Female	4	2	Legacy 27 mm	Complete	None	Aspirin 81 mg	L arm paresthesia	Continue aspirin monotherapy	-	No
3	Stroke	0 d	61	Male	1	0	Legacy 30 mm	Complete	DOAC	None	R MCA stroke	Aspirin monotherapy in peristroke period	Sludge and low flow in LAA	No
4	TIA	498 d	86	Female	4	4	FLX 24 mm	Complete	None	Aspirin 81 mg	Aphasia	Continue aspirin monotherapy	ASD present	No
5	TIA	339 d	83	Female	5	2	FLX 35 mm	Complete	None	Aspirin 81 mg	L arm paresis	Started clopidogrel and statin for small vessel disease	Severe mitral regurgitation	No

ASD = atrial septal defect; DOAC = direct oral anticoagulant; MCA = middle cerebral artery; other abbreviations as in Tables 1, 2, and 4.

### Bleeding

A total of 34 bleeding events occurred in 28 patients (17.4%) and were classified according to the BARC definitions. Some patients had two simultaneous bleeds (eg, gastrointestinal and genitourinary), while others had two separate bleeding events separated by time. Most bleeding events were type 2 (n = 18, 11.2%), whereas 7 cases (4.3%) were type 3a and 2 cases (1.2%) were type 3b. There were 2 cases (1.2%) of intracranial hemorrhage (type 3c) and 1 case (0.6%) of a fatal bleed (type 5b). The bleeding types were gastrointestinal (n = 19, 12.0%), genitourinary (n = 5, 3.1%), epistaxis (n = 5, 3.1%), intracranial (n = 2, 1.2%), (n = 2, 1.2%), and hemarthrosis (n = 1, 0.6%). Twelve bleeding events occurred on aspirin and DOAC (7.5%), 7 on aspirin monotherapy (4.3%), 7 on DOAC monotherapy (4.3%), three on aspirin and clopidogrel (1.9%), and one each for warfarin monotherapy (0.6%), non-steroidal anti-inflammatory monotherapy (0.6%), aspirin and non-steroidal anti-inflammatory therapy (0.6%), and triple therapy with aspirin, clopidogrel and a DOAC (0.6%). The first bleeding event occurred within 45 days in 14 patients (8.7%), between 45 and 180 days in 5 patients (3.1%), and after 180 days in 9 patients (5.6%).

### Arrhythmia recurrence

There were 157 patients (97.5%) who had follow-up beyond the 90-day blanking period. Of those, 63 (40.1%) had an arrhythmia recurrence. These patients had persistent AF (44.4%) at baseline, followed by paroxysmal (41.3%) and long-standing persistent (14.3%). Follow-up arrhythmia management included DCCV in 45 patients (28.0%), repeat ablation in 11 patients (6.8%), and AV node ablation in 2 patients (1.2%).

### Medication discontinuation

Of the 161 patients who successfully completed the procedure, 151 (93.8%) had their OAC successfully discontinued. Reasons for continuing OAC were elevated stroke risk (n = 5, 3.1%), venous thromboembolism (n = 2, 1.2%), minor leak (n = 1, 0.6%), lost to cardiology follow-up (n = 1, 0.6%), and periprocedural stroke (n = 1, 0.6%). Of the 161 patients, 53 (32.9%) were on a class 1 or class 3 anti-arrhythmic drug before the procedure. Of those 53 patients, 29 (54.7%) successfully discontinued anti-arrhythmic therapy during follow-up.

### Leak follow-up

Of the 40 patients with a PDL identified on initial follow-up imaging, 7 (17.5%) had repeat imaging. The mean time to the most recent repeat imaging was 590.7 ± 318.0 days with a median of 617.3 days (IQR: 315.8-750.9 days). Indications for repeat imaging included routine surveillance in 3 patients, valvular heart disease in two patients, bacteremia in one patient, and repeat ablation in one patient. There were four leaks that resolved and three leaks that persisted.

All persistent leaks occurred with the legacy device. One was a major leak (>5 mm) that was previously minor (≤5 mm) and occurred with the legacy WATCHMAN device. Of the four leaks that resolved, three were with the legacy WATCHMAN device, and one was with the FLX device.

### DRT follow-up

Both patients with DRT identified on initial follow-up imaging had repeat imaging. Both cases occurred in patients with the legacy WATCHMAN device. Both DRTs eventually resolved on extended anticoagulation upon reassessment with TEE imaging. DRT resolved in 185 and 377 days, respectively. None resulted in thromboembolic events. Anticoagulation was indefinitely discontinued for the patient whose DRT resolved on day 185. In contrast, the other patient who had heart failure and reduced ejection fraction (30%) and a high CHA_2_DS_2_-VASc score of 5 remained on apixaban 5 mg twice daily until DRT resolved. Subsequently, it was decided to maintain the patient on a low dose of apixaban 2.5 mg twice daily due to his significant risk factors.

## Discussion

We aimed to assess the safety and efficacy of combining AF ablation and LAAC in a single procedure. Specifically, we evaluated procedural success, periprocedural complications, thromboembolism, bleeding events, and arrhythmia recurrence. To our knowledge, this report represents the largest U.S. single-center patient series to date. This is in contrast to some of the existing studies, such as the series by Phillips et al (2018)^14^ that included 139 patients across 11 different centers internationally.

Our study provides a more robust evaluation of efficacy and safety utilizing this combined approach than previously reported and includes the newer generation WATCHMAN Flex device. In addition, it is essential to note that this is a high-risk patient population that may reflect the type of patients encountered in practice who are considered for the concomitant procedure, as they were older (mean age 72.1 years), predominantly with nonparoxysmal AF (52.8%), and at high risk for thromboembolism (mean CHA_2_DS_2_-VASC 4) and bleeding (mean HAS-BLED 2.8).

Both procedures have been studied individually, with overall favorable safety and efficacy profiles.^1^, ^2^, ^3^, ^4^, ^5^^,^^9^^,^^10^^,^^12^^,^^13^ However, combining both procedures carries the risk of additional adverse events and potential barriers to successful completion. The increased procedural time and manipulation of both the access site and within the atrium may predispose to higher rates of vascular complications and perforation with serious pericardial effusion. Acute physiological effects of ablation (edema, endothelial dysfunction, and atrial stunning) may result in unsuccessful procedures and DRTs, and the resolution of these changes may contribute to late PDLs and device embolization.

On the other hand, given the procedural similarities of AF ablation and LAAC, a concomitant procedure could potentially have significant benefits to the patient, including a shorter anticoagulation period and the risk of one instead of two procedures. It may also reduce health care resource utilization (anesthesia, imaging, and procedural supplies) resulting in cost savings.

In our study, performing concomitant AF ablation and LAAC procedures did not adversely impact the success rate of LAAC when compared to previously published data. The success rate was 87.3% with the legacy WATCHMAN device and 97.4% with the WATCHMAN FLX. These success rates are comparable to the results of trials evaluating LAAC alone. The PROTECT AF^10^^,^^11^ and PREVAIL^9^ trials demonstrated a success rate of 90.9% and 95.1%, respectively, whereas the PINNACLE FLX trial evaluating the newer generation WATCHMAN FLX showed a success rate of 98.8%.

The rate of perioperative complications in our study was relatively low. There were 2 cases (1.1%) of perforation with tamponade. When compared to the number of effusions requiring surgical repair in the PROTECT AF trial (1.6%) and the PREVAIL^9^ trial (0.4%), there appears to be no added risk of serious pericardial effusion with performing ablation and WATCHMAN implant concomitantly. The periprocedural stroke rate in our group was 0.6%, comparable to that in PROTECT AF^10^^,^^11^ (1.1%) and PREVAIL^9^ (0.4%). There were no cases of device embolization in this cohort, compared to the two patients in PROTECT AF^10^^,^^11^ and two patients in PREVAIL.^9^ There was one case of AV fistula in our group, compared to one case in the PREVAIL^9^ group. Finally, there were 5 cases (2.8%) of pericarditis in our patient population, comparable to or less than what is reported in the literature (up to 10.2% in a prior study of pulmonary vein isolation ablation^15^). It is important to note that the current study reflects our early experience with the concomitant LAAC and ablation procedure. A recent report by Piccini et al (2023)^16^ showed reassuring safety outcomes of the combined procedure (1.8% for any major adverse event at 45 days and 0.2% for pericardial effusion), indicating that procedural safety will continue to improve with increased experience.

Our study highlights the potential efficacy and safety of a concomitant procedure in managing thromboembolic events without imposing additional downstream risks or device-related complications. Over a mean follow-up duration of 553 days, we observed a total of 5 thromboembolic events (two strokes and three TIAs). Based on these results, the risk of stroke was 1.7% at 1 year and 4.6% at 2 years, which is notably lower than the anticipated stroke risk in a cohort with a mean CHA_2_DS_2_-VASc score of 4, predicting an annual stroke risk of 3.8% at 1 year and 7.6% at 2 years. In comparison to Phillips et al, which reported a 1.09% risk of stroke at 2 years vs our study with a 4.6% risk at 2 years, our cohort included a sicker population (older patients [72.1 vs 64.2 years] years, with a higher mean CHA_2_DS_2_-VASc score [4 vs 3.4], and more patients with persistent AF [52% vs 29%]). Moreover, while our study provides additional observational evidence supporting the short-term outcomes of the combined approach, the Phillips et al^17^ findings corroborate our observations, further underscoring the favorable long-term efficacy and safety profile of combining LAAC and ablation in a single procedure.

The rate of DRT was low at 1.3% (n = 2). This compares favorably with the rates seen in the Primary Outcome Evaluation of a Next-Generation Left Atrial Appendage Closure Device (PINNACLE FLX) trial (1.8%) and the PROTECT AF trial^10^^,^^11^ (5.7%). All DRTs resolved with anticoagulation.

There was a higher rate of minor device leaks in this cohort (26.3%) than previously reported in the PINNACLE FLX trial (17.2%). However, our cohort included the legacy WATCHMAN device, and two-thirds of these leaks were ≤3 mm. When considering the FLX device alone, the leak rate drops to 21.7%. This may be caused by the resolving procedural edema. No major leaks were reported on initial imaging, and one was identified during subsequent repeat imaging. This is similar to the findings of the PINNACLE FLX trial, and better than those reported in the PROTECT AF^10^^,^^11^ (7.5%).

Most patients had their OAC discontinued (93.8%), compared to the near 100% discontinuation rates in the PREVAIL,^9^ PROTECT AF,^10^^,^^11^ and PINNACLE trials. This likely reflects the nuanced and often difficult decisions patients and their providers face in clinical practice, who may opt to continue anticoagulation for added protection in those at the highest risk for thromboembolism so long as the risk of adverse bleeding events does not outweigh the benefits. Another explanation may be the uncertainty surrounding the severity of PDLs, in which case continued OAC allows ongoing protection while awaiting repeat imaging. Lastly, some patients had other indications for anticoagulation, such as venous thromboembolism.

Some concerns remain with the concomitant procedures. The size of the LAA can potentially change due to LAA ostial edema, changes in preload, or conversion to sinus rhythm. In the current study, we did not observe any significant changes in dimensions between pre- and post-ablation measurements. The operators chose the appropriate device based on the larger diameter measured pre- or post-ablation. However, based on TEE images (such as those in Figure 2), it may be reasonable to delay the LAAC portion if clinically appropriate when there is significant edema, defined as edema extending to the landing zone on the Coumadin ridge. No attempts were made to oversize the LAAC device in anticipation of ostial tissue edema post-ablation. In our experience, the landing zone is usually distal to the edematous area. Furthermore, the concern with increased DRT risk and subsequent thromboembolism remains a concern due to ablation edema-resultant endothelial dysfunction. In addition, we did observe a slightly higher rate of minor leaks than what has been previously reported. This may either be related to missed leaks at implant or, more likely, a result of the development of new small minor leaks after the procedure due to fluid shifts and/or continued remodeling.

### Study limitations

The retrospective and observational nature of the study does not allow for adequate elimination of bias and adjustment for confounding. Moreover, it was conducted at a large, quaternary center of considerable experience in cardiac care, with expert providers carrying out the procedure in a distinct patient population, which may affect the generalizability and external validity of the study. Additionally, the current analysis is based on results from a single center rather than a multicenter study, which further limits the generalizability of the results.

## Conclusions

Concomitant AF ablation and LAAC seems feasible, provided it is performed by an experienced operator, and significant edema at the landing zone is carefully monitored. It may provide a way of addressing arrhythmia burden, stroke risk, and bleeding risk simultaneously in appropriate patients. The anticipated Comparison of Anticoagulation With Left Atrial Appendage Closure After AF Ablation (OPTION) trial^18^ will hopefully expand our knowledge about the feasibility and safety of this procedure.

Perspectives **COMPETENCY IN PATIENT CARE AND PROCEDURAL SKILLS:** In patients with symptomatic AF who are identified as having both high thromboembolic and bleeding risk, concomitant AF ablation and LAAC appears to be safe and feasible and may provide a way of addressing arrhythmia burden, stroke risk, and bleeding risk simultaneously in a resource-efficient manner.

**TRANSLATIONAL OUTLOOK:** Concomitant AF ablation and LAAC may serve as a one-stop procedure for patients with symptomatic AF and high thromboembolic and bleeding risk.

## Funding support and author disclosures

Dr Wazni has served as a consultant for Biosense Webster and Boston Scientific. Dr Callahan has served as a consultant for Biotronics and Philips. Dr Santangeli has received speaking honorarium from Boston Scientific. Dr Rickard has received research grant from 10.13039/100008497Boston Scientific, 10.13039/100004374Medtronic, Ireland, and 10.13039/100000046Abbott. Dr Taigen has served as a consultant for Medtronic and Biosense Webster. Dr Jaber has served as a consultant for Boston Scientific and Pfizer. Dr Saliba has served as an advisory board member for Boston Scientific. Dr Kanj has received speaking honorarium from Boston Scientific; and has served as a consultant for Boston Scientific. All other authors have reported that they have no relationships relevant to the contents of this paper to disclose.
