# Supplementary material for: Outcomes of Concomitant Atrial Fibrillation Ablation and Left Atrial Appendage Closure: A Retrospective Single-Center Experience
Source: JACC Adv. 2024 Nov 14;3(12):101377. doi: 10.1016/j.jacadv.2024.101377 (PMC11609395; doi:10.1016/j.jacadv.2024.101377)
Supplement: Supplemental Table and Figures [file mmc1.docx]

**SUPPLEMENTAL APPENDIX**

Bleeding Academic Research Consortium 3 or Bleeding Academic Research Consortium 5 guidelines:

- *Type 1:* bleeding that is not actionable and does not cause the patient to seek an unscheduled performance of studies, hospitalization, or treatment by a health care professional; it may include episodes leading to self-discontinuation of medical therapy by the patient without consulting a health care professional.
- *Type 2:* any overt, actionable sign of hemorrhage (e.g., more bleeding than would be expected for a clinical circumstance, including bleeding found by imaging alone) that does not fit the criteria for type 3, type 4, or type 5 but does meet at least one of the following criteria: requiring nonsurgical, medical intervention by a health care professional; leading to hospitalization or increased level of care; or prompting evaluation.
- *Type 3a:* overt bleeding plus a hemoglobin drop of 3 to 5 g/dL* (provided the hemoglobin drop is related to bleed); any transfusion with overt bleeding.
- *Type 3b:* overt bleeding plus a hemoglobin drop of 5 g/dL (provided the hemoglobin drop is related to bleed); cardiac tamponade; bleeding requiring surgical intervention for control (excluding dental, nasal, skin, and hemorrhoid); bleeding requiring intravenous vasoactive agents.
- *Type 3c:* intracranial hemorrhage (does not include microbleeds or hemorrhagic transformation, does include intraspinal); subcategories confirmed by autopsy or imaging, or lumbar puncture; intraocular bleed compromising vision.
- *Type 4:* coronary artery bypass grafting-related bleeding; perioperative intracranial bleeding within 48 hours; reoperation after closure of sternotomy for the purpose of controlling bleeding; transfusion of 5 U of whole blood or packed red blood cells within a 48-hour period; chest tube output 2 L within a 24-hour period.
- *Type 5a:* probable fatal bleeding; no autopsy or imaging confirmation but clinically suspicious.
- *Type 5b:* definite fatal bleeding; overt bleeding or autopsy, or imaging confirmation.

Supplemental Table 1: Indications for concomitant procedure

| **Indication for concomitant procedure** | **#** | **%** |
| --- | --- | --- |
| Gastrointestinal bleeding | 51 | 28.7% |
| Elevated falls risk | 33 | 18.5% |
| History of intracranial hemorrhage | 13 | 7.3% |
| Anemia | 11 | 6.2% |
| Epistaxis | 11 | 6.2% |
| Genitourinary bleeding | 8 | 4.5% |
| Warfarin non-compliance | 5 | 2.8% |
| History of subarachnoid hemorrhage | 4 | 2.2% |
| Concomitant antiplatelet and non-steroidal anti-inflammatory use | 4 | 2.2% |
| Stroke while on oral anticoagulation | 4 | 2.2% |
| Severe thrombocytopenia | 3 | 1.7% |
| Severe ecchymoses | 3 | 1.7% |
| Medication side effect | 2 | 1.1% |
| Occupational hazard | 2 | 1.1% |
| Gynecologic bleeding | 2 | 1.1% |
| Hematologic malignancy | 2 | 1.1% |
| Spinal bleed | 2 | 1.1% |
| Labile INR | 2 | 1.1% |
| Medication interaction | 2 | 1.1% |
| Unspecified bleeding | 1 | 0.6% |
| Aortic aneurysm | 1 | 0.6% |
| Hereditary hemorrhagic telangiectasia | 1 | 0.6% |
| Bleeding via arteriovenous fistula | 1 | 0.6% |
| Recurrent intraocular procedures | 1 | 0.6% |
| Persistent left atrial appendage thrombus on oral anticoagulation | 1 | 0.6% |
| Recurrent orthopedic injections | 1 | 0.6% |
| Recurrent pericarditis | 1 | 0.6% |
| Hemarthrosis | 1 | 0.6% |
| Recurrent paracentesis | 1 | 0.6% |
| Recurrent head trauma | 1 | 0.6% |
| Warfarin related osteoporosis and fracture | 1 | 0.6% |
| Aortic dissection | 1 | 0.6% |
| Oral bleeding | 1 | 0.6% |

Supplemental Figure 1: Periprocedural complication rates

Supplemental Figure 2: Device seal on initial follow-up imaging stratified by device type.
